# Obstetric Accidents, Emergencies, and Operations

**Published:** 1897-06

**Authors:** 


**Obstetric Accidents, Emergencies, and Operations.** By L. Ch. Boisliniere, M.D. Pp. 381. Philadelphia: W. B. Saunders. 1896.

While physiology would have us believe that labour is a natural process, Dr. Boisliniere, a man evidently of great experience in everyday life, writes a fair-sized book dealing only with the accidents which may befall the pregnant and parturient woman, and the mischances which may happen to the unconscious traveller to independent existence.

In the first chapter the causes, diagnosis and treatment of abortion are explained briefly but fully. For stopping the hemorrhage Dr. Boisliniere recommends the tampon, which may "be left in place from eight to ten hours, and be replaced by a fresh tampon if necessary." In England, as Dr. Boisliniere duly notes, it is now taught that when there is complete placenta prævia and well-marked hemorrhage, expectant treatment ought to be discarded, and delivery had recourse to without delay, for the next hemorrhage may be very copious, and perhaps fatal. Dr. Boisliniere, however, prefers a more expectant plan; viz., the use of tampon, as recommended by Depaul and other French authorities. In accidental hemorrhage, which usually occurs late in pregnancy, it is not advisable to plug the vagina, for it may cause a considerable amount of blood to accumulate within the uterus, which may be large and also wanting in tone, and the result may be fatal. Immediately after considering retention of the placenta he speaks of atresia of the vagina and rupture of the uterus; but why the one very rare condition and the other most rare and dangerous accident should be treated as if they occurred after instead of during labour, it is difficult to understand. In fact, in the second portion of Part I. we meet with a want of method and order, and we find a heterogeneous list of complaints jumbled up together, without any very evident relation to one another as regards cause and effect, which may well confuse a young and inexperienced practitioner. It is, however, a great treat to read, near the end of Part I., the excellent chapter on puerperal convulsions. This chapter is a delightful contrast to those immediately before it as regards clearness, order, regular arrangement, and completeness, especially the last portion, under the head of treatment. One of the best remedies for puerperal eclampsia which our forefathers so firmly believed in and practised, is now gradually gaining its lost ground in America. The abandonment of such a remedy is thus commented on by Dr. Boisliniere:—"In America, discarding the teachings of the old masters—Dewees, Byford, Fordyce Barker, Hodge, Gross, as well as of Lusk—and of the whole English and French schools, and swayed by the modern German authorities, the lancet has almost entirely been laid aside. How many of those who read this book possess a lancet and have been taught how to use it? We do not bleed now because we do not know how to bleed properly." An illustrious school of accoucheurs like that of France has never lost faith in this remedy.

Part II. is very important, for it describes obstetric operations which are rendered necessary by mechanical obstructions to labour, arising from deformed pelvis and untoward positions of the child, all of which may require turning, the use of the forceps, embryotomy, and the *dernier ressort* of midwifery; viz., operations such as Cæsarean section and Porro's operation, laparotomy and symphyseotomy, &c. This part begins with a very able chapter on abdominal palpation, an assistance to early and correct diagnosis which is too much neglected in this country, but in America is appreciated at its true value, or even beyond it. The well-executed plates which illustrate this chapter are copied from photographs, representing an accoucheur clad in a sort of pinafore, with his shirt sleeves tucked up above his elbows, manipulating the naked abdomen of a woman who lies before him. These pictures are neither ornamental nor useful, for obstetric information of that kind is to be derived from the sense of touch and not of sight. All the obstetric manœuvres involved in version are well described. We think, however, that the rather elaborate chapter which treats of the "extraction of the child" would have come in better in that place if it had been preceded by remarks on natural delivery in cases where the breech, knees and feet originally present. In favourable cases of this kind the child may be delivered without let or hindrance, and without any interference on the part of the accoucheur. If, as frequently happens, such interference should be required, a few plain and concise directions should have been given how to effect this. In Chapter v., which may be considered as the second portion of Part II., and which includes all obstetric operations requiring instruments, the author begins with a good account of the forceps, and its advantages and disadvantages; and this is followed immediately by the "obstetrician's bag," which, as the book is intended for country practitioners, far removed from obstetric aid or advice, contains descriptions of all sorts of instruments and every kind of drug which may be required in the most unusual and difficult cases. It would have been far better, we think, if this had been inserted at the beginning of Part II., or, better still, of the book itself. It is, in fact, an illustration of the want of order and arrangement to which we have previously alluded. This, however, is made amends for by a capital description of various kinds of forceps, and the best mode of using each in exceptional cases. It should be mentioned that in America, when the forceps is used, the dorsal decubitus as employed in France and mostly on the Continent is generally preferred to the usual English position, with the woman lying on the left side. Chapter VI. deals with those formidable operations which require the knife. The account of all these operations is very well written, very interesting and well worth reading; but when the author tells us that his work is meant for a guide-book for a man who is "thrown upon his own resources," and when further on he speaks of the Cæsarean section, and says that "four assistants are necessary, and a nurse to receive the child!" we humbly suggest that these very interesting sections may possibly be a dangerous superfluity, and may tempt some surgical tyro to undertake operations which may easily, under the circumstances in which he is placed, become beyond his power. Besides, in the majority of cases requiring these desperate operations, there is generally ample time for due consideration and transporting the patient to some place where a good hospital would insure all the advantages of thoroughly qualified assistants. The last chapter of Part II. describes the operation of embryotomy and the instruments required for it.

Part III. includes accidents to the child during labour, diseases and abnormalities, monstrous children, fractured bones during birth, asphyxia neonatorum and umbilical hemorrhage, &c. This part is one of minor importance compared with the first two parts; but though short, it contains much that is very good, and deserves a longer notice than we are able to give it.

Like many of the American works we have seen lately, this book is handsomely got up by the publisher, and deserves much commendation for the excellence of its paper, the clearness of its type, and the great beauty of the numerous illustrations.

Since writing this review we have learned with great regret that Dr. Boisliniere died while his book was being printed. An appreciative notice of his long life and honourable career, accompanied by a portrait, may be seen in Vol. VIII. of the *Transactions of the American Association of Obstetricians and Gynecologists.*

